# Camera-based automated monitoring of flying insects in the wild (Camfi). II. flight behaviour and long-term population monitoring of migratory Bogong moths in Alpine Australia

**DOI:** 10.3389/finsc.2023.1230501

**Published:** 2023-09-13

**Authors:** Jesse Rudolf Amenuvegbe Wallace, David Dreyer, Therese Maria Joanna Reber, Lana Khaldy, Benjamin Mathews-Hunter, Ken Green, Jochen Zeil, Eric Warrant

**Affiliations:** ^1^ Research School of Biology, The Australian National University, Canberra, ACT, Australia; ^2^ National Collections & Marine Infrastructure, CSIRO, Parkville, VIC, Australia; ^3^ Lund Vision Group, Department of Biology, Lund University, Lund, Sweden; ^4^ School of Life and Environmental Sciences, The University of Sydney, Sydney, NSW, Australia; ^5^ College of Asia and the Pacific, The Australian National University, Canberra, ACT, Australia

**Keywords:** Camfi, Bogong moth, population monitoring, flight behaviour, insect conservation, navigation, migration, aestivation

## Abstract

**Introduction:**

The Bogong moth *Agrotis infusa* is well known for its remarkable annual round-trip migration from its breeding grounds across eastern and southern Australia to its aestivation sites in the Australian Alps, to which it provides an important annual influx of nutrients. Over recent years, we have benefited from a growing understanding of the navigational abilities of the Bogong moth. Meanwhile, the population of Bogong moths has been shrinking. Recently, the ecologically and culturally important Bogong moth was listed as endangered by the IUCN Red List, and the establishment of a program for long-term monitoring of its population has been identified as critical for its conservation.

**Methods:**

Here, we present the results of two years of monitoring of the Bogong moth population in the Australian Alps using recently developed methods for automated wildlife-camera monitoring of flying insects, named Camfi. While in the Alps, some moths emerge from the caves in the evening to undertake seemingly random flights, filling the air with densities in the dozens per cubic metre. The purpose of these flights is unknown, but they may serve an important role in Bogong moth navigation.

**Results:**

We found that these evening flights occur throughout summer and are modulated by daily weather factors. We present a simple heuristic model of the arrival to and departure from aestivation sites by Bogong moths, and confirm results obtained from fox-scat surveys which found that aestivating Bogong moths occupy higher elevations as the summer progresses. Moreover, by placing cameras along two elevational transects below the summit of Mt. Kosciuszko, we found that evening flights were not random, but were systematically oriented in directions relative to the azimuth of the summit of the mountain. Finally, we present the first recorded observations of the impact of bushfire smoke on aestivating Bogong moths – a dramatic reduction in the size of a cluster of aestivating Bogong moths during the fire, and evidence of a large departure from the fire-affected area the day after the fire.

**Discussion:**

Our results highlight the challenges of monitoring Bogong moths in the wild and support the continued use of automated camera-based methods for that purpose.

## 1 Introduction

**Figure 1:**
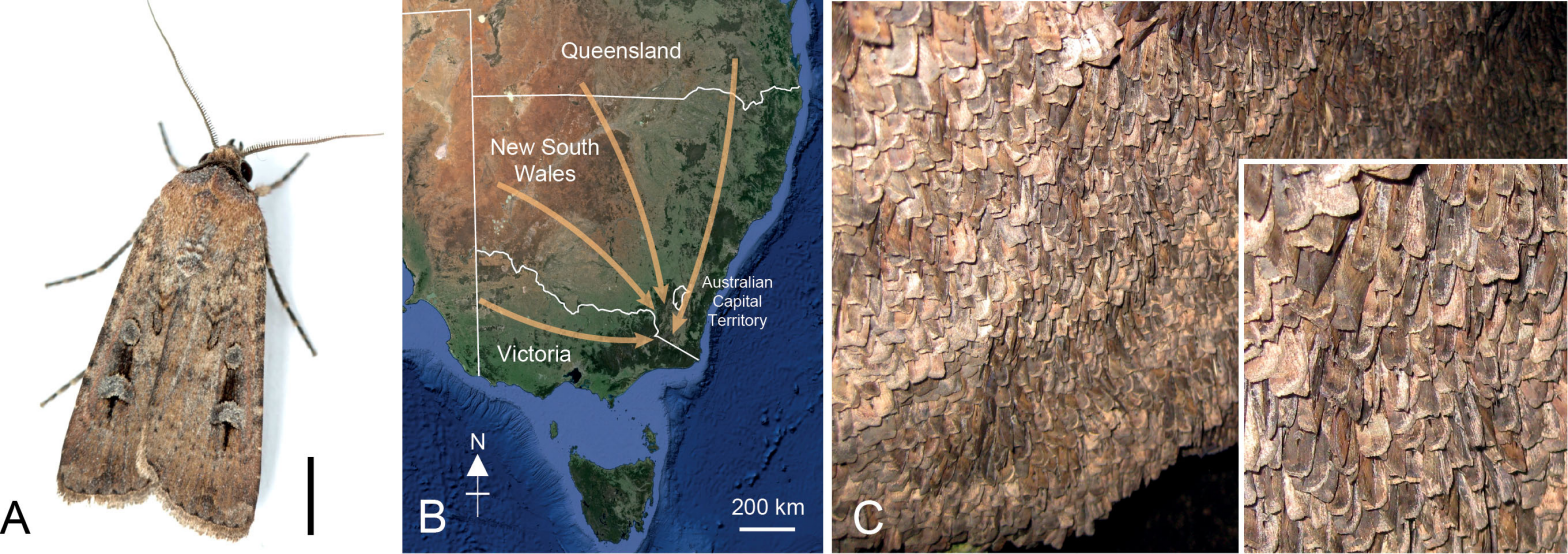
The Bogong moth. **(A)** A male Bogong moth. Scale bar = 5 mm. Photograph: Ajay Narendra, Macquarie University **(B)** Likely migratory routes (*arrows*) of moths during spring to alpine regions in southeast Australia. Autumn migration occurs in the reverse directions. **(C)** Around 17,000 moths/m^2^ aestivate on the walls of specific caves in the Australian Alps for up to 4 months before making the return migration. Photographs: Eric Warrant.

The Bogong moth *Agrotis infusa* is well known for its remarkable annual round-trip migration from its breeding grounds across eastern and southern Australia to its aestivation sites throughout the high mountain areas of New South Wales, Victoria, and the Australian Capital Territory, where it forms aggregations numbering in the millions (**Figure 1**; reviewed by 1). Bogong moth aestivation—equivalent to hibernation during the summer—was first reported during the 19^th^ century (2, 3), but the summer assemblages of moths have been known by Australian Aboriginal people in the areas surrounding the Australian Alps for millennia (4–7). Despite this long-standing knowledge, neither Bogong moth migration nor the function of the summer aestivation were scientifically detailed until the 1950s (8, 9).

In recent years, increasing efforts have been made to understand the migration of the Bogong moth from a neuroethological perspective (e.g. 1, 10–12), particularly with respect to how Bogong moths navigate. However, an open question remains as to what the proximate triggers for Bogong moth migration are (1). As well as being interesting in its own right, the answer to this question is rapidly becoming critical to the conservation of the unique Australian Alpine ecosystem, which accommodates many species that rely on the annual influx of nutrients brought by the Bogong moth migration (13–15). Concerningly, an estimated 200-fold reduction in the Bogong moth population was observed between the 2016–2017 and 2017–2018 summers, following a slow, but consistent decline since the early 1980s (16–18). This has led to the recent listing of the Bogong moth as endangered on the IUCN Red List (19).

The question of what proximate cues trigger Bogong moth migration is complex and difficult to solve. Behavioural experiments are laborious, and indeed, to our knowledge, a behavioural paradigm to measure the timing of a Bogong moth’s migration in response to controlled stimuli has yet to be developed. It therefore seems prudent to make quantitative measurements of Bogong moth migratory timing in the wild. This will enable us to determine what proximal factors are correlated with the behaviour, which will greatly assist in narrowing the search-space for future experimentation.

Useful progress to this end has been made through long-term monitoring of migrating insects using vertical radar deployed on the Bogong moth migratory route (e.g. 20). However, reliable monitoring of Bogong moths in their breeding grounds remains an unsolved challenge (21). At the end of their spring migration, a number of methods have been used to monitor Bogong moths close to their aestivation sites, including light trapping (15, 21), light beam surveys, (22), aestivation site surveys (17, 23), ski surveys of Bogong moth carcasses on the snow (17), and fox scat surveys (24, 25). Each of these methods have their idiosyncrasies, and are to varying degrees laborious, limiting their utility for large-scale long-term monitoring programs, such as the 100-site Bogong moth monitoring program recommended by Wintle et al. (21).

An opportunity for non-invasive monitoring of Bogong moths at their summer aestivation sites is presented by a peculiar behaviour exhibited by the moths. During their summer aestivation, Bogong moths emerge from their caves shortly after sunset and perform seemingly random flights (1, 9). Although these flights are only undertaken by a small fraction of the moths at any particular site, they are enough to fill the air with densities probably reaching dozens per cubic metre (Wallace, personal observations). We propose that by measuring the relative intensity of these evening flights with sufficient precision, we can accurately model the relative abundance of Bogong moths at the aestivation sites over a summer season, and over multiple years.

In addition to being useful for enabling long-term non-invasive monitoring, the summer evening flights of Bogong moths are themselves an interesting unresolved phenomenon, worthy of investigation. The purpose of these flights is unknown, although observations have been made of Bogong moths using them to visit water to drink (1, 9). The flights must serve an important function, or they would surely be strongly selected against, since taking flight is energetically costly and exposes moths to predation by bats. It is reasonable to hypothesise that these evening flights play a role in in the most conspicuous aspect of the Bogong moth’s life history: its migration. However, with the exception of a single drinking and feeding experiment (9), we are aware of no previous attempt to measure or characterise this behaviour beyond *ad-hoc* qualitative description.

In this paper, we present the results of two observational studies into the summertime behaviour of Bogong moths in the Australian Alps using inexpensive wildlife cameras.

In the first study, we deployed wildlife cameras pointing towards the sky, taking still images, outside Bogong moth aestivation sites for two study periods spanning December 2019 to March 2020, and September 2020 to April 2021, respectively. We show that by monitoring the sites for the full span of the Bogong moth aestivation season, we can infer the arrival and departure dates of the moths from those sites. Moreover, for the first time we are able to quantitatively analyse the evening twilight flight activity of the aestivating Bogong moths (described by 8) over the entire duration of the summer, providing strong preliminary evidence for weather being an important driver of flight behaviour – and by extension, migratory behaviour – in the moth. In addition, we deployed a camera inside a Bogong moth aestivation cave during the 2019–2020 summer, pointing towards a cluster of aestivating moths. While the camera was deployed in the cave, a bushfire came within 1 km of the site, and we were able to observe the effect the nearby bushfire on the assemblage of moths.

In the second study, we deployed multiple video cameras over a single night (18^th^ February 2021) along two elevational transects below the summit of Mt. Kosciuszko, a known Bogong moth summer habitat. In this study, we demonstrate that evening flights are not random but are systematically oriented in directions relative to the azimuth of the mountain summit. The observation of directed flights may provide clues as to their purpose, which could include calibration of their navigational machinery (reviewed by 26–28) or searching for more favourable sites to continue their aestivation (13).

Our results, and indeed the method we have developed to obtain them, may be of interest to land managers and conservationists who seek to measure the ongoing effects of management practices on the declining population of the endangered Bogong moth, and to monitor it more generally.

## 2 Study 1: population monitoring

### 2.1 Methods (study 1)

The methods employed in this study (known collectively as Camfi) are described in detail in the preceding paper in this journal (29). The Camfi package is freely available from https://github.com/J-Wall/camfi. Weather data were obtained from weather stations close to the camera sites (Thredbo AWS 071032, 30; Mount Ginini AWS 070349, 31; Cabramurra SMHEA AWS 072161, 32).

Camfi was used to monitor the activity of migratory Bogong moths arriving at, aestivating in, and departing from their summer range in the Australian Alps, using still images obtained from off-the-shelf wildlife cameras (see 29 for a full description of the method).

#### 2.1.1 Camera placement and settings

Study sites were selected for their proximity to known Bogong moth aestivation sites. Cameras were placed at an upward angle, such that they had a clear view of the sky, but that rainwater would not pool on their lenses. Depending on the terrain, a suitable mounting place was chosen making use of small tripods, tension straps, or wedging the camera between boulders. The precise placement is not particularly important, however it was noted so that in subsequent years the same placement could be used. Cameras were set to take images using their timelapse function, with one capture every 5–10 minutes (detection counts are normalised based on image frequency during analysis).

**Figure 2:**
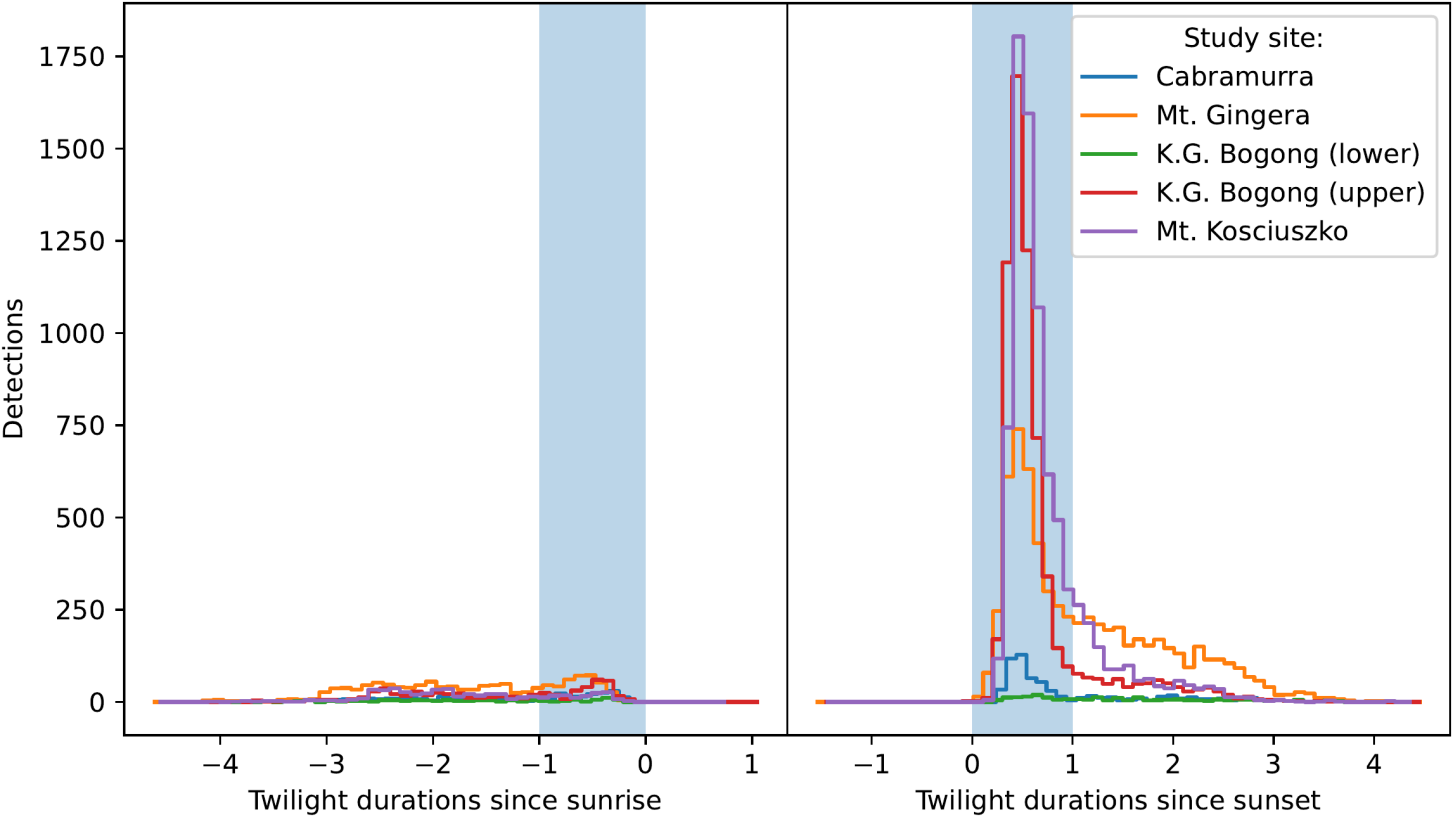
Total number of moth observations by time relative to sunrise (*left*, scaled by the duration of morning twilight) does not show peak in activity during morning twilight (*blue shaded region*), with the slight exception of K.G. Bogong (upper site) and Mt Gingera, which show small peaks. Total number of moth observations by time relative to sunset (*right*, scaled by the duration of evening twilight) shows a peak in activity during evening twilight (*blue shaded region*) across all study sites.

In the first study season (2019–2020), cameras were placed outside a Bogong moth aestivation site in a boulder field near the summit of Mt Kosciuszko, NSW, and outside two aestivation sites near the formerly unnamed peak now known as Ken Green Bogong (referred to in this paper as K.G. Bogong), near South Rams Head, NSW. In the second study season (2020–2021), a site near the summit of Mt. Gingera, ACT/NSW, which has been subject to a number of previous studies (6, 9, 23) was added. Pilot data obtained in November 2019 from a boulder field near Cabramurra, NSW, were also included for certain analyses (see **Figure 2**).

During the 2019–2020 season, a single camera was also placed inside the aestivation cave on K.G. Bogong, facing towards a cluster of aestivating Bogong moths (referred to as the “observation cluster”). This camera was not used for automated annotation, although occasionally flying moths were seen inside the cave. By the end of the season this camera had been flooded and was no longer usable.

#### 2.1.2 Image annotation

A total of 118,361 still images of the sky were obtained during this study. These images were automatically annotated using Camfi. Wingbeat frequencies of each annotation were also measured by analysing the wingbeat patterns visible in the images of moving moths using Camfi. For further analyses, the automatically obtained annotations were filtered by prediction score, wingbeat signal-to-noise ratio (SNR; as measured by Camfi), and wingbeat frequency (see 29). In particular, annotations with prediction scores less than 0.8, wingbeat SNR outside of the range [1,50] , or wingbeat frequency outside of the range [27,78] Hz were excluded.

#### 2.1.3 Data analysis

Of primary interest is the relative daily abundance of flying Bogong moths at the study sites and across the study period. We measured this by counting moth detections occurring during evening twilight, noting the number of images collected at a given site during the evening twilight of a given day as the exposure variable.

Various daily abiotic factors were regressed against counts of Bogong moths detected by Camfi during evening twilight (elevation, maximum and minimum and range of temperature, maximum wind speed, twilight duration, study year, relative humidity (9 am), latitude and rainfall). Before performing the regression, the Pearson correlations between each pair of factors were calculated, and highly correlated factors were removed using a greedy recursive algorithm. The algorithm proceeded by selecting the most highly correlated pair of factors, then removing the factor in the pair which was less well correlated with the evening detection count. The algorithm terminates when no pair of factors had a Pearson $R^{2}$ greater than a specified threshold, which we set to 0.3. The remaining factors were then jointly regressed against the evening detection count using a Poisson regression with image count as the exposure variable.

These analyses make the implicit assumption that repeat detections of the same individuals do not occur or are not important. While the validity of this assumption is difficult to precisely assess, we believe it is reasonable given the extremely low marginal probability of detecting a particular moth flying in the vicinity of a site (the cameras only view a small portion of the sky, and a typical photo exposure of ~0.1 s only represents about 0.03% of the time interval between exposures). This issue may become more relevant when taking images with a short interval, but as we see in the flight behaviour study below, Bogong moth evening flights are typically highly directed, so it is hard to imagine a moth waiting in a camera's field of view for long enough to get duplicate detections.

### 2.2 Results (study 1)

**Figure 3:**
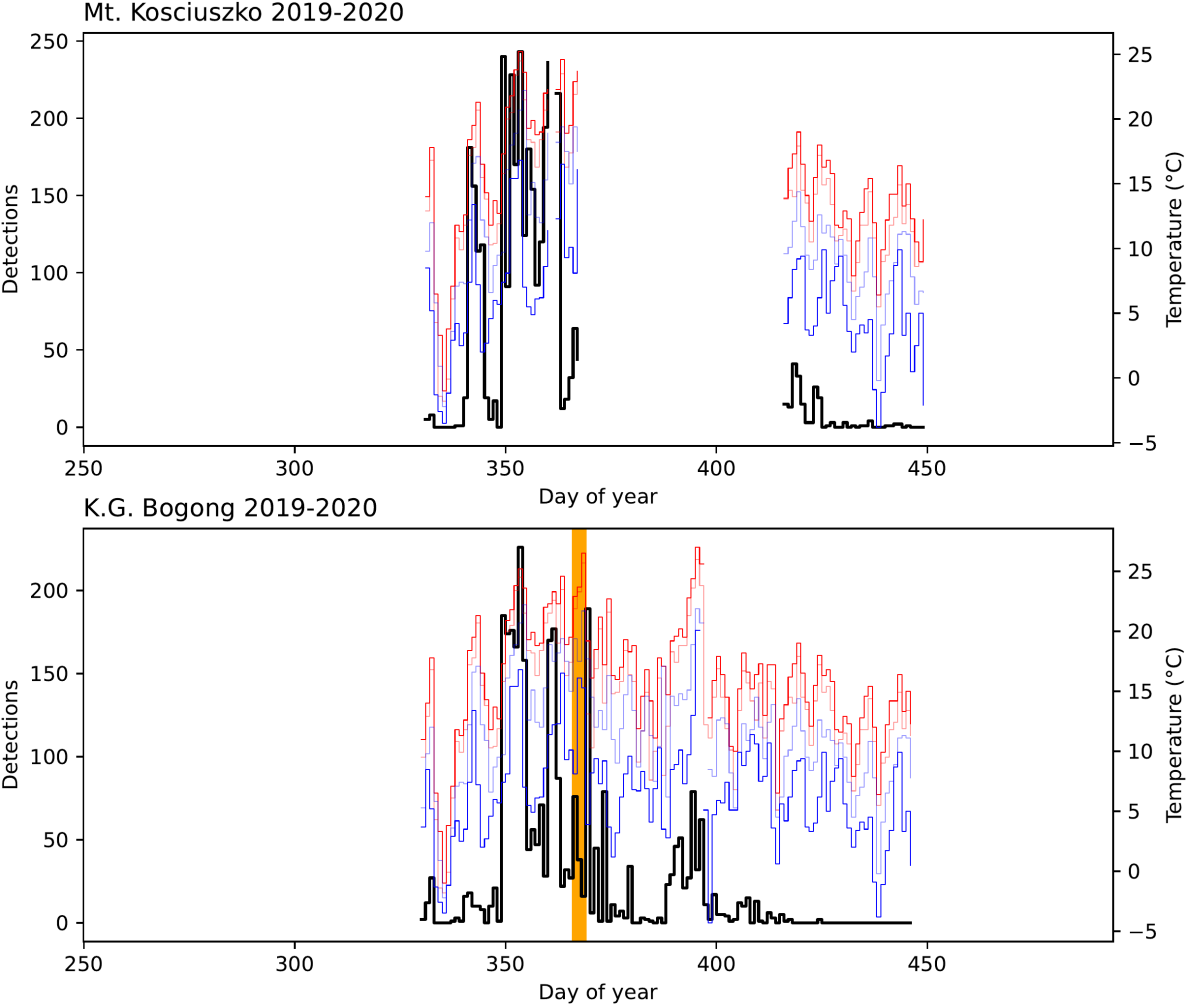
Number of Bogong moth detections (*black*) for each study day in the 2019–2020 summer season outside Bogong moth aestivation sites on Mt. Kosciuszko and K.G. Bogong, NSW, shown with daily temperatures recorded at Thredbo Top Station (30): maximum (*red*), minimum (*blue*), 9 am (*light blue*), and 3 pm (*light red*). Data are missing for a portion of the season at the Mt. Kosciuszko site due to a camera malfunction. *Orange span* indicates a bushfire event which occurred 1 km SW of K.G. Bogong (the fire did not reach the site, although there were high levels of smoke in the air which would have entered the site).

**Figure 4:**
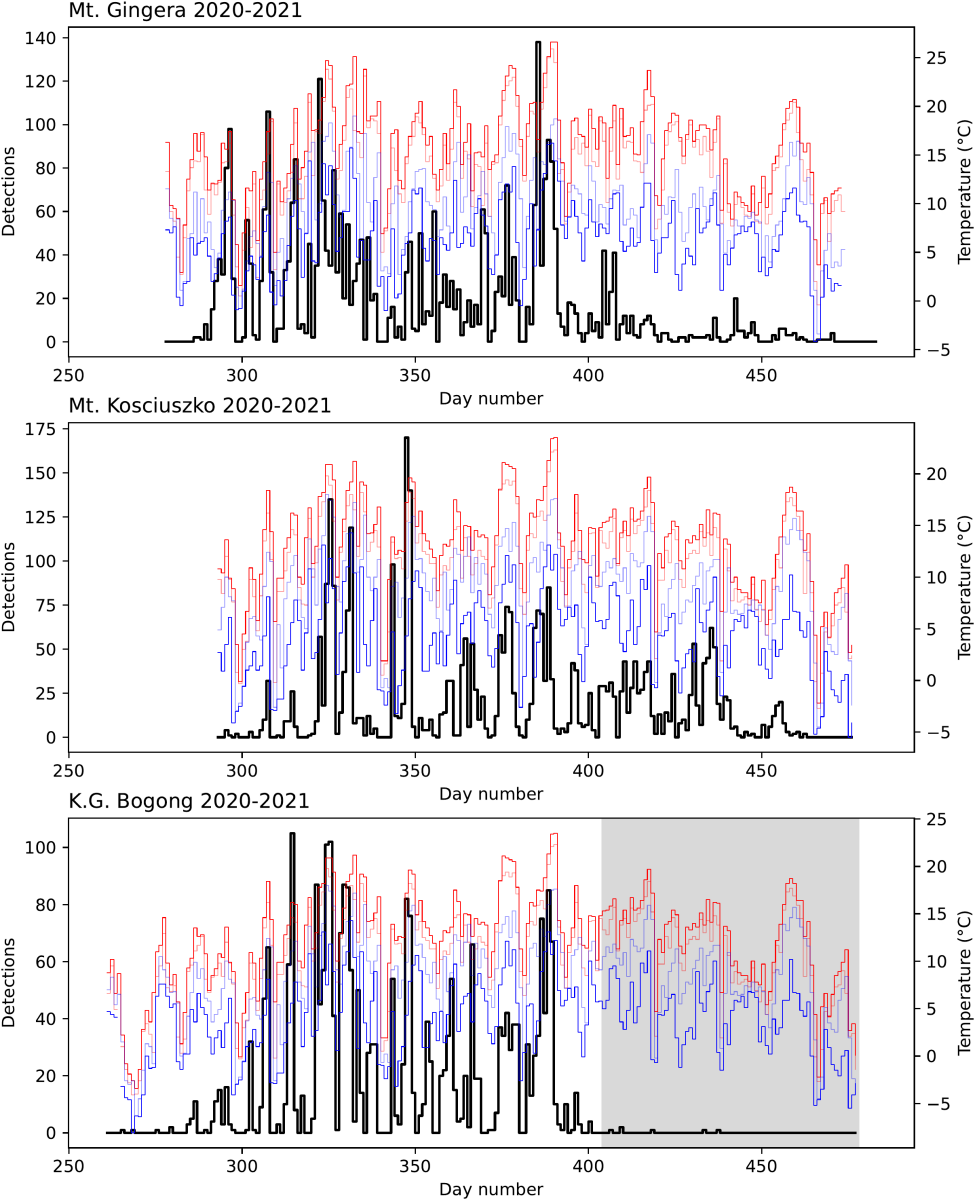
Number of Bogong moth detections (*black*) for each study day in the 2020–2021 summer season outside Bogong moth aestivation sites on Mt. Gingera, Mt. Kosciuszko, and K.G. Bogong, NSW, shown with daily temperatures recorded at Mt Ginini and Thredbo Top Station (30, 31): maximum (*red*), minimum (*blue*), 9 am (*light blue*), and 3 pm (*light red*). Shaded region on lower plot indicates period where camera had fallen from its mount, reducing its view of the sky by about half.

Strong peaks in activity were observed during evening twilight across all study sites (**Figure 2**, *right panel*), with a long tail of activity seen later into the night, although pronounced peaks were not seen during morning twilight (**Figure 2**, *left panel*). It should be noted that day-time detections are unreliable with the current version of Camfi so the close to zero detections during the day in **Figure 2** should not be overinterpreted (although based on our observations very few Bogong moths fly during the day). Evening twilight detection counts were highly variable, but clearly show that Bogong moths departed from the lower elevation sites (Mt. Gingera and K.G. Bogong) earlier in the season than from higher elevation sites, i.e. Mt. Kosciuszko (**Figure 3** for the 2019–2020 summer and **Figure 4** for the 2020–2021 summer). The camera placed at the K.G. Bogong site fell from its mount towards the end of the season (**Figure 4**, *lower panel, shaded region*), reducing the camera’s view of the sky by about half, however it appears that the majority of moths had already left the area by the time this happened.

#### 2.2.1 Predictors of activity

**Figure 5:**
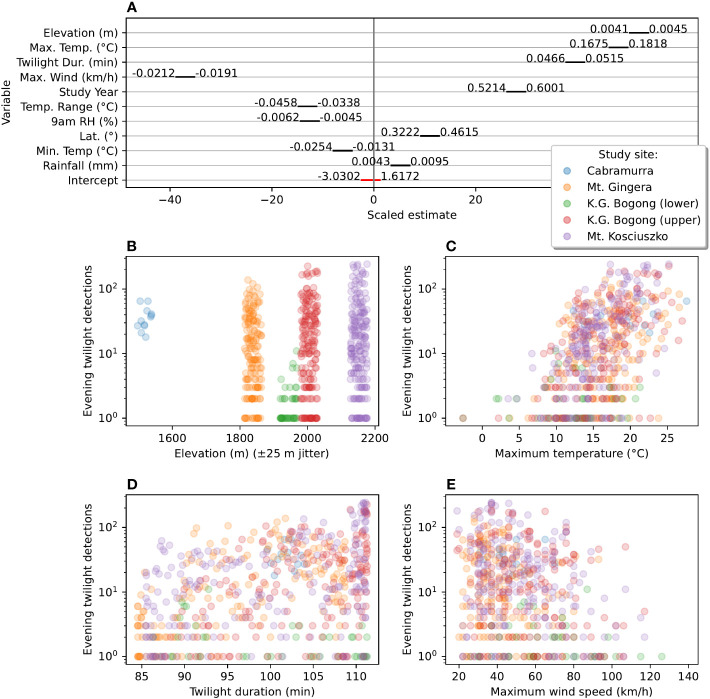
Effect-sizes and plots of number of detections during evening twilight against significantly associated abiotic factors. **(A)** Scaled estimates of effect size of abiotic factors on Bogong moth evening flight intensity (as measured by number of Camfi detections) from a mixed-effect Poisson generalised linear model of detections against these factors. *Black bars* show 95% confidence interval of estimates (scaled by effect size). *Values* to either side of *black bars* represent bounds of 95% confidence interval in the units of the respective factor (corresponding to the gradient of the regression, in that dimension). *Negative values* indicate that increases in the value of the factor lead to a decrease in moth counts (and *positive values*, the opposite). **(B)** Scatter plot of detections per evening twilight by elevation. Random fluctuation (jitter) is applied to elevation to increase readability. **(C)** Scatter plot of detections per evening twilight by daily maximum temperature. **(D)** Scatter plot of detections per evening twilight by duration of evening twilight. **(E)** Scatter plot of detections per evening twilight by daily maximum wind gust speed. Points in **(B–E)** are *coloured* by study site, as per study-site key (*right, towards top*).

A total of ten abiotic factors were found to be significantly correlated with evening twilight counts of flying Bogong moths (**Figure 5A**): elevation, maximum and minimum and range of temperature, maximum wind speed, twilight duration, study year, relative humidity (9 am), latitude and rainfall. A greater number of moths were observed at higher elevation sites (**Figure 5B**) and when twilight duration was longer (i.e. in the middle of summer, **Figure 5D**). Study year was also positively correlated with moth counts, suggesting that the Bogong moths were more abundant in the 2020–2021 summer than in the 2019–2020 summer. The most important weather factors were daily maximum temperature (which had a positive effect on moth counts, **Figure 5C**) and maximum wind speed (which had a negative effect on moth counts, **Figure 5E**). Note that very few Bogong moths were observed flying on days which had maximum temperatures lower than 10°C (**Figure 5C**). Daily temperature range, relative humidity (measured at 9 am on the morning prior), and daily minimum temperature were negatively correlated with moth counts, while latitude and rainfall were positively correlated with moth counts. Scatter plots of all covariates in our model are shown in **Figure S1**, and residuals of the fitted model are shown in **Figure S2 (Supplementary Materials)**.

#### 2.2.2 Arrival and departure of Bogong moths

During the 2020–2021 summer season, the cameras were placed at the aestivation sites in September, before the Bogong moths had arrived, and removed in April, after they had left, so the data contain information regarding the arrival and departure dates of the Bogong moths. For example, at Mt. Gingera, the first Bogong moth was detected on the 13^th^ October (**Figure 4**, *top panel, day 286*). At K.G. Bogong, this date was 11^th^ October (**Figure 4**, *bottom panel, day 284*). A few detections were made prior to this date, however upon inspection these were found to be false positives caused by rain. The first detection of a Bogong moth at Mt. Kosciuszko was on 22^nd^ October (**Figure 4**, *middle panel, day 295*) although cameras were only placed there on 20^th^ October, so it is possible that some moths had arrived earlier. However, snow was still present on the ground in front of the aestivation site on Mt. Kosciuszko until it melted on 23^rd^ October (Wallace, personal observation), so if there was an earlier arrival, it was probably only by a few days.

**Figure 6:**
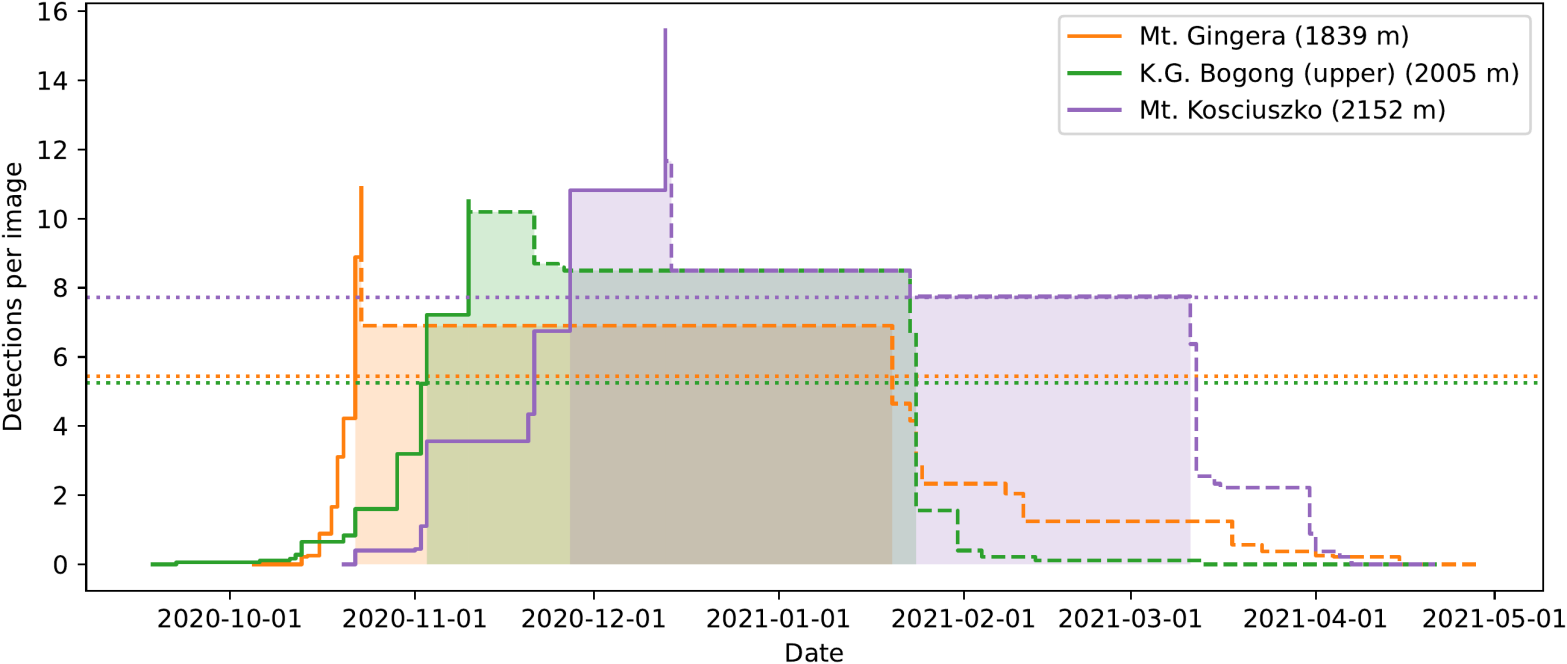
A simple heuristic model of the arrival and departure of Bogong moths to summer aestivation sites applied to data obtained from automated camera monitoring in the summer of 2020–2021. *Solid lines*: Cumulative maximum detections per image, plotted until the date that absolute maximum is reached, for the respective location. This roughly models the arrival of moths to the location. *Dashed lines*: Reverse-cumulative maximum of detections per image, plotted from date of absolute maximum, for the respective location. This roughly models the sum of departure and mortality of moths from the location. *Dotted lines*: Show half of the maximum detections per image for respective location (for calculating median date of arrival and departure). Dates after median date of arrival, and before median date of departure for each location are *shaded*. Elevations shown are of the camera placement, rather than the summit elevations of the mountains.

While records of earliest arrival are interesting, they are also subject to substantial noise, since the marginal probability of a particular moth being detected at all is very small. Earliest arrivals are also not necessarily representative of the predominant behaviour in the population. Therefore, we would like to use the detection data across the entire season to model the arrival and departure of the majority of the population. To do this, we propose a simple heuristic model of the evolving relative abundance of evening-flying Bogong moths in an area, as the moths arrive at—and later depart from—the area (**Figure 6**). This model is based purely on detection data, and is independent of weather factors, etc.

The model separates the aestivation of Bogong moths at a particular site into two phases; arrival and departure. In the arrival phase (**Figure 6**, *solid lines*), the relative abundance of aestivating moths is modelled by the cumulative maximum of the mean number of detections per image over all preceding evenings. The arrival phase ends on the day where this value reaches its maximum across the entire summer. The departure phase (**Figure 6**, *dashed lines*) is modelled similarly, this time using the reverse-cumulative maximum.

An obvious set of descriptive statistics arise; namely, median date of arrival and median date of departure. These are shown for each study site in the 2020–2021 season in **Figure 6** (*shaded areas*) and in **Table 1**. A clear signal of Bogong moths arriving at higher elevation aestivation sites later than lower elevation sites is present (**Figure 6**, *solid lines*; **Table 1**). This trend appears to also apply to departures, albeit slightly less clearly (**Figure 6**, *dashed lines*; **Table 1**).

**Table 1:** Median date of arrival ( $A_{1/2}$ ) and departure ( $D_{1/2}$ ) of Bogong moths from aestivation sites during 2020–2021 summer.

Date	Mt. Gingera (1839 m)	K.G. Bogong (2005 m)	Mt. Kosciuszko (2152 m)
Mt. Gingera $A_{1/2}$ (2020-10-22)	14.0°C (std = 2.0°C)	11.2°C (std = 1.7°C)	9.8°C (std = 1.7°C)
K.G. Bogong $A_{1/2}$ (2020-11-03)	14.0°C (std = 2.5°C)	14.0°C (std = 4.1°C)	12.7°C (std = 4.1°C)
Mt. Kosciuszko $A_{1/2}$ (2020-11-27)	20.1°C (std = 2.4°C)	17.3°C (std = 1.9°C)	16.0°C (std = 1.9°C)
Mt. Gingera $D_{1/2}$ (2021-01-20)	18.4°C (std = 2.2°C)	16.1°C (std = 0.9°C)	14.7°C (std = 0.9°C)
K.G. Bogong $D_{1/2}$ (2021-01-24)	24.3°C (std = 1.6°C)	20.7°C (std = 2.3°C)	19.4°C (std = 2.3°C)
Mt. Kosciuszko $D_{1/2}$ (2021-03-11)	16.4°C (std = 1.4°C)	14.4°C (std = 1.5°C)	13.0°C (std = 1.5°C)

Elevations shown are of the camera placement, rather than the summit elevations of the mountains. Temperatures are 3-day average maxima calculated across the 3 days preceding the date listed (inclusive), from the nearest weather station (30) assuming an adiabatic lapse rate of 9.1°C/1000 m elevation (33).

To assess whether temperature could explain the later arrival and departure of Bogong moths at higher elevations, we calculated the 3-day average maximum temperature at each location in the lead-up to the median arrival and departure (**Table 1**). Notably, temperatures were relatively high at lower elevation sites (Mt. Gingera: 20.1°C, K.G. Bogong: 17.3°C) in the days leading up to the median *arrival* date at the higher-elevation Mt. Kosciuszko site (2020-11-27; **Table 1**). Also, temperatures in the lead-up to median *departure* dates at the lower elevation sites were relatively high, respectively (Mt. Gingera: 18.4°C, K.G. Bogong: 20.7°C), while pre-departure temperatures at Mt. Kosciuszko were comparatively cool (13.0°C; **Table 1**).

#### 2.2.3 Impact of January 2020 bushfires

On January 4^th^ 2020, a major bushfire which had been burning in the area in the preceding few days (**Figure 3**, *orange span*) came within 1 km of the K.G. Bogong site. Despite the thick smoke, Bogong moths were seen flying outside their aestivation cave (**Figure S3, Supplementary Materials**). The following day, a large number of flying Bogong moths were detected, presumably indicating a departure of a portion of the moths from the site (**Figure 3**, peak to the right of *orange span*).

**Figure 7:**
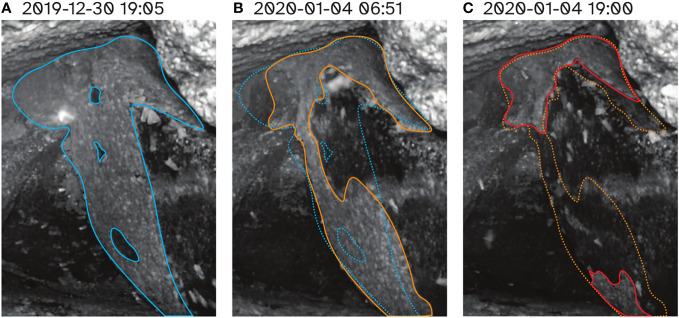
Progression of cluster of aestivating Bogong moths in cave on K.G. Bogong over the worst few days for Kosciuszko National Park during the 2019–2020 bushfire season. **(A)** Prior to bushfire. Cluster is outlined with *solid blue trace*. **(B)** Morning of 4^th^ January 2020, the day which saw the bushfire come within 1 km of the site. Cluster of Bogong moths is outlined with *solid orange trace*. Trace from **(A)** is overlaid for comparison (*dotted blue trace*). **(C)** Evening of 4^th^ January 2020. Cluster of Bogong moths is outlined with *solid red trace*. Trace from **(B)** is overlaid for comparison (*dotted orange trace*). Times shown are Australian Eastern Daylight Time (AEDT; UTC+11:00).

A reduction in the number of aestivating Bogong moths on K.G. Bogong during the bushfire was reflected by the observation cluster inside the cave, which dramatically reduced in size over the course of the fire (**Figure 7**). Notably, a significant portion of this reduction happened during the day (**Figures 7B, C**), despite Bogong moths typically being night-active. The remaining cluster (**Figure 7C**) did not change much during the following few weeks before the camera was flooded on 20^th^ January.

## 3 Study 2: evening twilight flight behaviour

### 3.1 Methods (study 2)

The video-tracking functionality of Camfi was used to track individual Bogong moths through consecutive frames of a video sequence captured by off-the-shelf wildlife cameras. Video sequences were captured throughout a single night by 10 cameras arranged along two approximately linear transects below the summit of Mt Kosciuszko, NSW (5 cameras per transect). Camfi was used to analyse these sequences and thereby determine the direction of displacement of Bogong moths as they travelled through the air.

#### 3.1.1 Camera placement and settings

**Figure 8:**
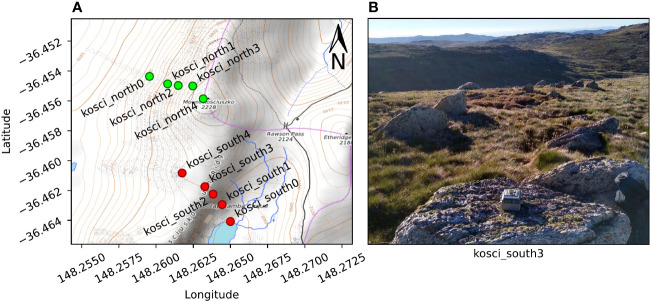
Cameras were placed on two transects on the slopes of Mt Kosciuszko, NSW. **(A)** Map of camera locations. *Contour lines* show 10 m changes in elevation. The transects were kosci_south (*red*), on the south-eastern slope towards Lake Cootapatamba and kosci_north (*green*), on the north-western slope, below the summit. **(B)** Example placement of camera at kosci_south3 location. Map data available under the Open Database Licence at openstreetmap.org. ^©^ OpenStreetMap contributors, SRTM. Map tiles credit: ^©^ OpenTopoMap (CC-BY-SA).

A total of ten cameras (BlazeVideo, model SL112) were placed in two transects (5 cameras per transect) below the summit of Mt Kosciuszko, NSW, on the afternoon of 18^th^ February 2021, and collected the following morning. The first transect, which we call kosci_south, was placed on the south-eastern slope, running from the shore of Lake Cootapatamba up to the Kosciuszko South Ridge, and ranging in elevation from 2046 m to 2151 m. The second transect, which we call kosci_north, was placed on the north-western slope below the summit, with five cameras ranging in elevation from 2050 m to 2220 m. The positions of each camera are shown in **Figure 8A**. The kosci_north1 and kosci_north2 locations were both within 10 m of known Bogong moth aestivation sites.

The cameras were placed such that their lenses pointed up into the sky, and the compass orientation of each camera was noted so that analysis of flight direction could be performed. **Figure 8B** shows an example of the placement of one of the cameras. The cameras were set to take an image, along with a 5 s video clip every 30 s for the duration of the evening. Illuminance of the clear sky was recorded at multiple time points during evening twilight, near the kosci_north2 location (**Figure 8A**) using a digital luxmeter (Hagner, model E4-X).

#### 3.1.2 Computational analyses

Flying Bogong moths were detected and tracked in the 5 s video clips using Camfi. Track directions were modelled with the orientation models described by Schnute and Groot (34) using the CircMLE R package (35). Maximum likelihood models were selected using Akaike’s information criterion (AIC, 36).

At approximately 20:30 Australian Eastern Daylight Time (AEDT; UTC+11:00) the external cameras switched to night mode and started using their infra-red flash. Video clips taken before this time were omitted from analysis as it was found that detection was unreliable for video clips taken in day mode.

### 3.2 Results (study 2)

**Figure 9:**
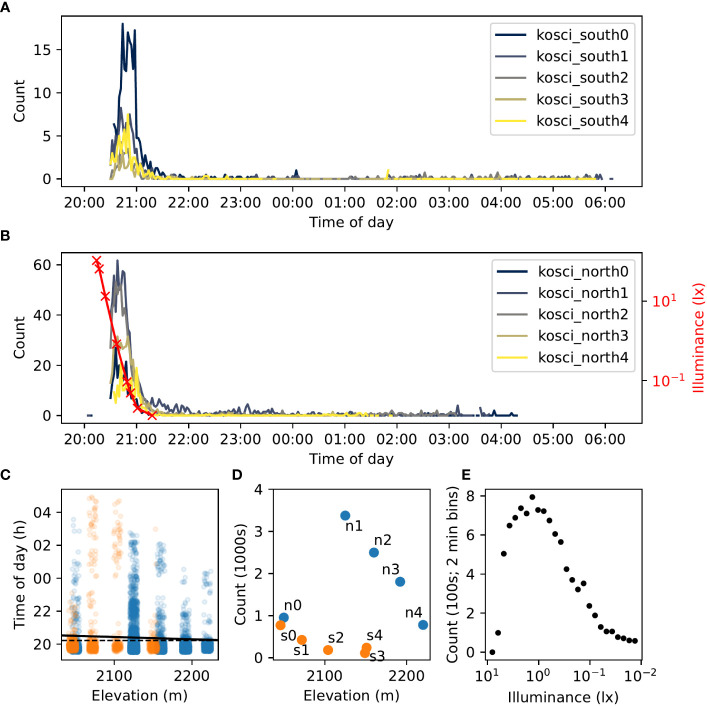
Summary of detections of flying insects on two transects of Mt. Kosciuszko on 18^th^–19^th^ February 2021. **(A, B).** Time series of counts of flying insect trajectories detected at each location in kosci_south **(A)** and kosci_north **(B)** transects. Counts have been smoothed by taking the average over 2 min bins. Illuminance readings recorded from the sky close to the kosci_north2 site are shown in **(B)** (*red trace*). **(C)** Time of each detection by elevation. Jitter (random fluctuation) is applied to elevation values to assist readability. A linear regression of time against elevation is shown by the *solid black line* ( $R^{2}=0.0065$ , slope = −5.59 s/m). Despite the small effect size, the slope is statistically significant (Wald test, $p=1.8\times 10^{-17}$ ; null “zero slope” hypothesis is indicated with the *dashed black line*). **(D)**. Total number of detections at each location by elevation. Location names are labelled “n0” = “kosci_north0”, “s3” = “kosci_south3”, etc. Points in **(C, D)** are marked according to transect; *blue* = kosci_north, and *orange* = kosci_south. **(E)**. Total detection count in 2-minute bins (pooled across all locations) plotted against illuminance (log-linearly interpolated from recorded measurements; **(B)**, (*red trace*). Measurements taken between 20:28 and 21:18 are included.

A total of 6,515 night-mode video clips were recorded, and from these 11,147 flying insects were detected. The vast majority of these are likely to be Bogong moths, as we observed a large number of them (and no other species) flying close to our vantage point near kosci_north2 throughout the evening. Sky illuminance varied from 106.5 lx to 0.0132 lx over the course of evening twilight (**Figure 9B**, *red trace*).

#### 3.2.1 Activity levels

A strong peak in activity was observed during evening twilight at all sites on both transects (as seen in our still-image experiments from the aestivation caves: see **Figure 2**). Activity plummeted just before 21:00 AEDT, coinciding with the end of nautical twilight, but continued at a low level throughout the night (**Figures 9A, B**). Outdoor illuminance dropped from about 1 lx during the activity peak to below 0.1 lx after activity had plummeted (**Figure 9E**). Of the 11,147 total flying insect detections, 8,589 (77%) occurred before 21:00 AEDT (from 576 video clips), and 10,163 (91%) occurred before 21:30 AEDT (from 1,176 video clips; coinciding with the end of astronomical twilight). This agrees with previous observations of Bogong moth flight activity exhibiting large peaks during evening twilight (1, 9). The activity peak was most pronounced at the kosci_north1 and kosci_north2 locations (**Figure 9D**), presumably owing to the proximity of these locations to known Bogong moth aestivation sites.

We also observed a statistically significant—albeit extremely weak—correlation between time of detection and elevation (Wald test, $p=1.8\times 10^{-17}$ , linear regression $R^{2}=0.0065$ , slope = −5.59 s/m; **Figure 9C**, *black line*). This corresponds to a delay in detections of roughly 16 minutes from the highest site (kosci_north4; 2220 m) to the lowest site (kosci_south0; 2046 m). We could tentatively take this as an indication that the bulk of the moths are moving downhill, although from this analysis alone, we are unable to disentangle that hypothesis from the hypothesis that moths at lower altitudes merely emerge from (and/or return to) their aestivation crevices later than higher-altitude moths. Nonetheless, evidence for a slight downhill movement during our single night of observations comes from analyses of flight orientation, the topic to which we turn next.

#### 3.2.2 Evidence of orientation behaviour

Displacement is, of course, not the only way to measure movement. We can also measure its derivative with respect to time; namely, velocity (the combination of direction of displacement, which we call “track direction”, and speed). In our case, we are only interested in the track direction of flights, which conveniently our method measures.

**Figure 10:**
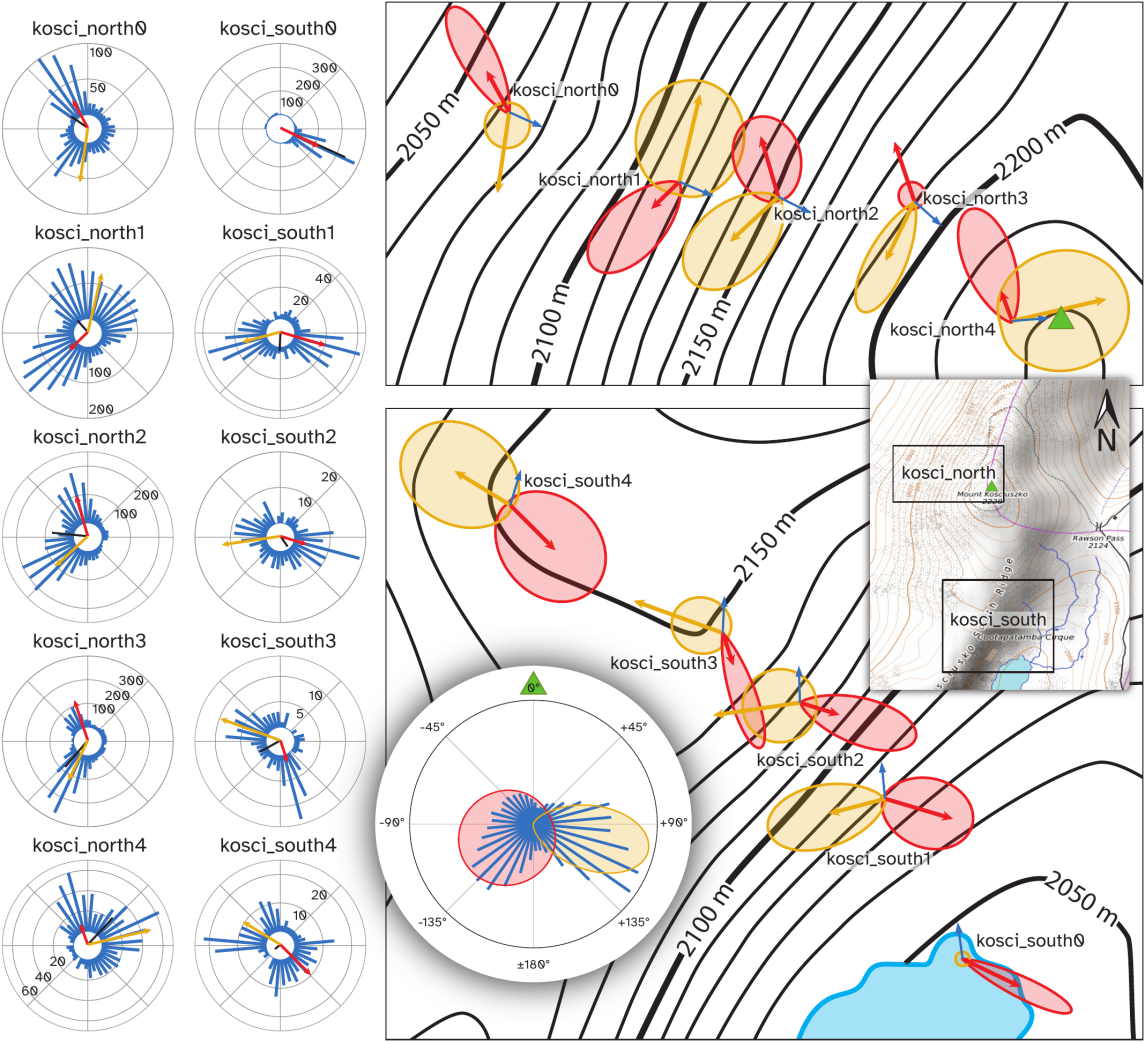
Distribution of flying insect track directions over the course of the evening of 18^th^ February 2021, by location. Left panel: *Blue bars* show histograms of track directions for the given location, with scale (counts) indicated on circular axes. *Black bars* show mean vector of all detections at the respective location. *Red arrows* show direction and weight of the first component of a bimodal von Mises distribution for the respective location, computed using the CircMLE R package (35) and *yellow arrows* show the second component. Where only a *red arrow* is shown, the data were better explained by a unimodal distribution. Top-right panel: *Arrows* from the left panel, with von Mises probability density functions of each component also plotted. Plots are placed at their respective camera locations on a map of the kosci_north transect. *Blue arrows* show the azimuth of the summit of Mt. Kosciuszko from the respective locations. The *green triangle* shows the summit of Mt. Kosciuszko. Bottom-right panel: Follows the conventions of the top-right panel, showing kosci_south transect. Inset, right: Map of Mt. Kosciuszko showing locations of top-right and bottom-right panels. The *green triangle* shows the summit of Mt. Kosciuszko. Circular inset, bottom: Histogram (*blue bars*) of flight track directions relative to the azimuth of the summit of Mt. Kosciuszko (*green triangle*) for all detections across all locations, shown with probability density functions of components of a bimodal von Mises model of the data (*red and yellow regions*).

Flight track directions at each respective site in both transects showed significant departures from uniform circular distributions (**Figure 10**), as determined by Moore’s modified Rayleigh tests ( p<0.05 for all locations, 37). Furthermore, mean track directions from each pair of locations were significantly different from each other, as determined by pairwise Mardia-Watson-Wheeler tests ( p<0.05 for each pair, 38). Thus, the flights of the Bogong moths were directed, and the direction of flight depended on location. This is not surprising, however it is ethologically relevant, since directed movement requires behavioural control in response to external stimuli (39).

Generally speaking, the distributions of flight track directions at each location were bimodal (**Figure 10**; **Table S1**) and the two modes were not separated by 180° (i.e. the bimodality of the directions was not a result of axially-directed flight). There was one notable exception to this trend, with moths detected at kosci_south0 showing a unimodal south-easterly flight track direction tendency (**Figure 10**, southernmost site; **Table S1**). Trends in flight track direction which were seen during nautical twilight (i.e. before 21:00 AEDT) continued throughout the night, albeit with a much lower density of moths (**Figure S4, Supplementary Materials**).

Flight track directions relative to the azimuth of the summit of Mt. Kosciuszko also clustered bimodally (pooled across all locations, confirmed by AIC-based maximum-likelihood model selection, **Table S2**; distributions shown in **Figure 10**, *right panels* and *circular inset*). The respective means of the two components of a bimodal von Mises model, fit to flight track directions relative to the azimuth of the summit were -118° (SD: 80.2°) and +103° (SD: 28.8°) (**Figure 10**, *circular inset*). As both of these are greater (in absolute terms) than 90°, the Bogong moths were, in aggregate, moving away from the summit. And since there is no higher point in Australia than the summit of Mt. Kosciuszko, the moths were also moving somewhat downhill, on paths which would take them around the mountain.

## 4 Discussion

Our results clearly and quantitatively demonstrate that the summer flights of Bogong moths, first described by Common (9), occur predominantly during evening twilight and occur throughout the Bogong moth’s entire summer aestivation. Our results also show that the intensity of these flights is modulated by daily weather factors, with Bogong moths favouring warmer evenings with lower wind speeds for flying. Moreover, it is clear from our video analyses that during these nightly excursions the Bogong moths exhibit orientated flight behaviour.

### 4.1 Discussion of study 1: population monitoring

#### 4.1.1 The possible roles of weather and climate on moth numbers

The influence of weather on animal migration *in general* has been well studied (see review by 40). Broadly, our results from Study 1 agree with those of previous studies comparing weather with flight activity of migratory insects. Namely, wind speed has a negative effect on flight activity (e.g. 41), and temperature has a positive effect (e.g. 42, 43).

For simplicity’s sake, we chose to use a relatively simple linear model relating moth counts to various abiotic factors. If long-term monitoring of Bogong moths continues using our camera-based method, and an increasing number of years of data become available, more complex models will become more appropriate. For example, we have modelled our study year as having a linear effect on moth counts. For a two-year study, this is valid, however when additional years are added, this should be changed to a random effect (or perhaps an effect depending on annual climactic factors, depending on the research question).

Our survey of three known Bogong moth aestivation sites over the summer of 2020–2021 shows that occupation of higher elevation aestivation sites by Bogong moths occurs later in the season than lower elevation sites. There are three possible explanations for the later arrival dates at higher elevations: 1) The higher sites are blocked by snow (or are otherwise unsuitable) earlier in the season but are open later to allow occupation by later arrivals of Bogong moths coming directly from the breeding grounds, 2) Bogong moths from lower sites move higher as summer progresses, or 3) a combination of 1 and 2.

Blockage of high-elevation aestivation sites by snow (possibility 1) would certainly prevent Bogong moths from occupying those sites, but this does not appear to be a satisfactory explanation for the delay we observed, as most of the remaining snow near the highest site (Mt. Kosciuszko) melted on 23^rd^ October 2020, more than an entire month prior to the median arrival of Bogong moths at this location. Additionally, sub-zero temperatures have been recorded in occupied Bogong moth aestivation sites (17), so it seems unlikely that low temperatures alone would have *prevented* Bogong moths from migrating directly to high-altitude sites early in the season.

On the other hand, high temperatures do appear to be a reasonable explanation for Bogong moths *avoiding* lower elevation sites, which could motivate movement to higher elevations (possibility 2 or possibility 3). The median arrival date at the highest elevation site (Mt. Kosciuszko) coincided with lower elevation sites experiencing 3-day average maximum temperatures above 16°C (**Table 1**). Incidentally, Green et al. (17) estimate that 16°C is the maximum temperature (inside a cave) that permits aestivation. Interestingly, 3-day average maximum temperatures leading up to the median *departure* dates at Mt. Gingera and K.G. Bogong were 18.4°C and 20.7°C at those locations, respectively (i.e. well above 16°C; **Table 1**). However, the 3-day average maximum temperature at Mt. Kosciuszko leading up to its median *departure* date was lower, at just 13.0°C. Therefore, it could be the case that departures from the Mt. Gingera and K.G. Bogong sites were motivated by high temperatures (and resulted in movements to higher elevations), while departures from Mt. Kosciuszko were motivated by temperatures falling (to 13°C), indicating the approaching autumn, thus triggering the return migration to the breeding grounds.

Notably, possibility 2 is also supported by previous results from fox scat surveys (25), which showed a departure of Bogong moths from sub-alpine areas into alpine areas as the summer progressed. However, properly disentangling each of these possibilities requires observations of the movements (or lack thereof) of Bogong moths between elevations during the summer, after their arrival in the mountains. Such observations could be made using a similar method to that used in this study, with cameras deployed in elevation transects on a single mountain.

Our simple model of Bogong moth arrival and departure is robust to periods of evening-flight inactivity (e.g. due to unfavourable weather conditions for flight), and follows naturally from the following two heuristics: 1) the maximum relative density of flying moths in the vicinity of an aestivation site is representative of the relative abundance of aestivating moths in the area, and 2) most Bogong moths arrive at and leave from the vicinity of an aestivation site on relatively few nights (so evening flights with lower relative density are generally station-keeping movements, rather than migrations to other aestivation sites or returning to the breeding grounds). As with the model for relating evening flights to abiotic factors, the arrival and departure model could perhaps be extended to include—depending on the research question—the effects of other factors, such as daily weather and annual climate.

#### 4.1.2 The effects of fire and smoke

Fire appears to have a pronounced effect on assemblages of aestivating Bogong moths. We observed a marked reduction in the size of our observation cluster of aestivating Bogong moths during the day that a bushfire came close to the site (but did not affect it directly). Presumably this was mediated by smoke entering the cave and disturbing the moths. Interestingly, from flight data, we observed (what we assume to be) a large departure of Bogong moths from a bushfire-affected area the day *after* the fire, and thus the day after the marked reduction in the size of the observation cluster. It could be that the reduction of the observation cluster on the day of the fire was caused by Bogong moths falling from their perch on the cave wall, but remaining inside the cave, rather than perishing or departing that day. These moths could then have departed the following day when conditions outside were less dangerous.

#### 4.1.3 Population monitoring and conservation

In recent years, especially since their dramatic population crash in 2017 (16, 17), Bogong moths have enjoyed increased attention from those interested in their conservation, and in the conservation of the Australian Alpine ecosystem more generally. In particular, the need for the implementation of a long-term monitoring program for Bogong moths for the ongoing conservation of the Australian Alpine ecosystem has been identified (21).

The high level of variability in counts we observed across each night of our study highlights the importance of regular measurements throughout the summer for such a monitoring program. Not only does the proportion of Bogong moths flying on a given night depend on the weather, but the Bogong moth population also moves between aestivation sites over the course of the summer, complicating the interpretation of sparse data collected from infrequent light-trapping surveys, particularly when these surveys do not simultaneously collect counts from multiple locations.

Ideally, a long-term monitoring program for Bogong moths would collect counts of moths every day at every study site from mid-September until mid-May, completely covering the Bogong moth aestivation season. For a large-scale program with many study sites, light trapping or similarly labour-intensive approaches would be a costly undertaking. Conversely, a comparatively “hands-off” and non-invasive approach (i.e. not involving trapping), such as the automated camera-based monitoring method used in this study, could be relatively easily and inexpensively scaled, without the need for a large team of dedicated light-trappers. For instance, by replacing the wildlife cameras used in this study with permanent solar-powered and Internet-connected camera stations, the data acquisition portion of a large-scale Bogong moth monitoring program could be completely automated, with visits to study sites only needed for placement, maintenance, and eventual retrieval of the equipment. Such a program would produce an incredibly rich and informative dataset for ongoing efforts to model the dynamics of the Bogong moth population, and their migration to and from the Australian Alps.

### 4.2 Discussion of study 2: Bogong moths have oriented evening flight behaviour

#### 4.2.1 Which sensory cues might Bogong moths use to orient?

It is clear from our video analyses of Bogong moth flights during a single late summer evening (Study 2), that these moths were orientated in flight (**Figure 10**). However, what is not immediately clear is *how* they were orienting themselves. We know from laboratory assays of orientation behaviour that Bogong moths are able to orient themselves relative to visual landmarks in conjunction with the Earth’s magnetic field (11) and celestial cues (Dreyer et al., in prep.). Another possible source of directional information is wind, which is known to be used to control flight direction in another species of migratory noctuid moth, *Autographa gamma* (44). It is likely that Bogong moths also possess this ability.

Cues from celestial objects and the Earth’s magnetic field would be roughly the same across all study locations, given the small geographical area covered by the study (all locations were within 1.2 km of each other). Therefore, these cues alone could not explain the differences in the distributions of flight track directions observed across camera locations (**Figure 10**, *left panel*).

Wind speeds at 3 pm, 18^th^ February, and 9 am, 19^th^ February 2021 were moderate to fresh—4 ms^-1^ easterly and 5 ms^-1^ north-northwesterly, respectively (recorded at Thredbo Top Station, circa 4.6 km from the summit of Mt. Kosciuszko; 30, 31). These wind speeds are similar to the likely airspeed of a motivated Bogong moth and could have an important impact on their resultant track direction, especially for high-flying moths. The speed and direction of wind can be modulated by topography, so it is possible that wind varied between the study locations, although this was not measured. Thus, we cannot rule out the possibility that wind could explain the observed differences in flight track directions across locations. However, bimodal distributions of flight track directions would be hard to explain with wind alone.

We can say with certainty that the terrestrial visual panorama varies greatly from one study location to the next, especially between the two transects, which are on opposite sides of the highest point in the mountain range. Dreyer et al. (11) showed Bogong moths orienting themselves relative to the azimuth of an abstraction of the silhouette of a mountain peak (namely, a black triangle on a white background, above a black horizon). Mountains are striking visual landmarks in the Australian Alps where Bogong moths spend their summer. It is therefore reasonable to predict that wild Bogong moths, exhibiting directed flights in their summer range, might fly in directions relative to mountain peaks.

Indeed, we found evidence for this hypothesis by finding that flight track directions relative to the azimuth of the summit of Mt. Kosciuszko – Australia’s highest peak (2228 m) and the closest peak to all study locations in both transects – clustered bimodally (pooled across all locations: **Figure 10**, *circular inset*). This means that moths presumably fixed their flight heading by holding the azimuth of the summit at a constant obtuse angle with respect to their direction of travel, leading to trajectories that would resemble portions of outward logarithmic spirals centred on the summit, when viewed from above.

#### 4.2.2 Why do Bogong moths make evening twilight flights?

Our analyses of the rich dataset produced by our new method have so far told us *what* the Bogong moths were doing (moving in a particular somewhat downhill direction) and have provided us with a robust hypothesis for *how* they were doing it (flying relative to the summit of Mt. Kosciuszko, in an outward spiral centred on the peak). What remains to be answered is, *why* do Bogong moths behave this way? Indeed, why do they take flight almost every evening throughout the summer—a decidedly non-dormant activity—when they are supposedly aestivating?

We can identify two possible reasons for the evening flight behaviour we have observed. Firstly, the somewhat downhill direction of Bogong moth flight trajectories may indicate that the moths are actually beginning their return migration to their breeding grounds and leaving the high peaks for lower elevations. The date of our video observations was February 18^th^ 2021, and we know from our long-term monitoring data that the bulk of the Bogong moths had already left the lower elevation aestivation sites of Mt. Gingera and Ken Green Bogong by mid-February 2021, and that numbers on Mt. Kosciuszko were declining in that month (see **Figure 6**). So it is reasonable to conclude that the return migration had begun.

Secondly, the spiralling nature of their flights around the mountain (rather than flying in a straight line), may be connected to calibration of the moths’ internal compasses prior to departure from the mountains. The flights only occur just after sunset, so the flying moths would be able to see the azimuth of the sunset, as well as the polarized light pattern in the sky, which are both extremely stable compass cues. Similarly, they could be using their magnetic sense to perceive the Earth’s magnetic field (11), an even more stable compass cue. Meanwhile, the moths could be taking snapshots of the terrestrial—and possibly celestial—panorama (as suggested for dung beetles, 45), which they could later use to help them navigate at the start of their return migration to their breeding grounds, akin to learning flights performed by other insects (46). It is very likely that these various compass cues are integrated together and that evening flights are used by Bogong moths to calibrate multi-sensory internal compasses which they eventually use for their return migration. Such multi-sensory compass calibrations are thought to be performed by migratory songbirds (reviewed by 27, 28).

An open question is how the directed flight behaviour of the Bogong moths develops over the summer. We performed this study on a single night towards the end of the season, but what happens earlier in the season or when the weather is different? This indicates an obvious future direction for this research, which is to perform long-term monitoring (i.e. each night across a summer season) using transects—or a grid—of cameras in video mode. This may require a modification to the equipment used, as when set in video mode the battery life of the cameras is severely curtailed compared with the still image setting that we used for long-term monitoring presented above.

## 5 Conclusions

In this paper, we have shown that our new methods (29) – that enable us to inexpensively make ethological observations of wild insects in a high-throughput, quantitative, and completely non-invasive manner – provide an excellent tool for long-term monitoring of insect populations and their movements. In our case study of the endangered Bogong moth *Agrotis infusa*, we have continuously recorded their nightly flight activity in the mountains over two Australian summers and simultaneously quantified their flight behaviour at several alpine locations over the course of a single night. The data we have obtained have not only shed light on the nature and purpose of the mysterious nightly flights of the Bogong moth but has demonstrated the efficacy of our camera-based methods for long-term monitoring of insect populations in the service of conservation.

Our results have also generated an interesting and plausible hypothesis relating to the previously unexplained summer evening flight behaviour of Bogong moths, namely that the evening flights serve a specific navigational purpose. In particular, these flights might be used by Bogong moths to calibrate their internal compasses by integrating directional information provided by the azimuth of the setting sun, the polarisation pattern in the sky, the geomagnetic field, and the visual panorama.

## Data availability statement

The datasets presented in this study can be found in online repositories. The names of the repository/repositories and accession number(s) can be found below: https://doi.org/10.5281/zenodo.5242596 https://doi.org/10.5281/zenodo.4950570 https://doi.org/10.5281/zenodo.4971714 https://doi.org/10.5281/zenodo.5039891 https://doi.org/10.5281/zenodo.4972022 https://doi.org/10.5281/zenodo.5040011 https://doi.org/10.5281/zenodo.5040018 https://doi.org/10.5281/zenodo.6583127.

## Ethics statement

Ethical review and approval was not required for the study on animals in accordance with the local legislation and institutional requirements.

## Author contributions

EW, JZ, DD, and JW conceived the project. JW, TR, BM-H, DD, EW, and KG performed the fieldwork. KG provided essential knowledge of the locations of Bogong moth aestivation sites. TR, LK, BM-H, and JW performed the manual annotations of images of flying moths. JW and DD analysed the data. JW, EW, DD, and JZ interpreted the results. JW wrote the first draft of the manuscript, with input from EW. All authors contributed to the article and approved the submitted version.
