# Supplementary material for: Camera-based automated monitoring of flying insects in the wild (Camfi). II. flight behaviour and long-term population monitoring of migratory Bogong moths in Alpine Australia
Source: Front Insect Sci. 2023 Sep 13;3:1230501. doi: 10.3389/finsc.2023.1230501 (PMC10926487; doi:10.3389/finsc.2023.1230501)
Supplement: Supplementary file 1 [file DataSheet_1.pdf]

frontiers

Supplementary Material

Camera-based automated monitoring of flying insects in the wild (Camfi). II. Flight behaviour and long-term population monitoring of migratory Bogong moths in Alpine Australia

Wallace, J.R.A.[*], Dreyer, D., Reber, T., Khaldy, L., Mathews-Hunter, B., Green, K., Zeil, J., Warrant, E.J.[*]

*** Correspondence:** Jesse Wallace: jesse.wallace@csiro.au, Eric Warrant: eric.warrant@biol.lu.se

S1 Supplementary Figures

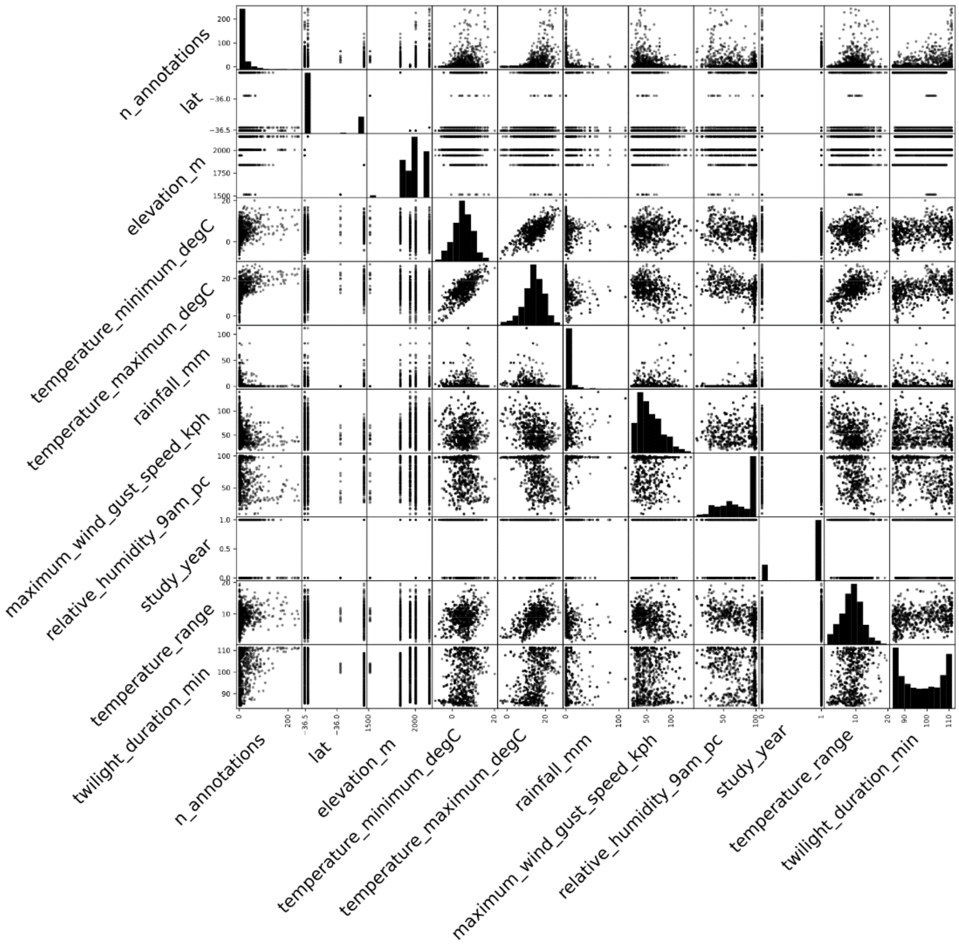

Figure S1. Scatter matrix of Bogong evening twilight flight covariates.

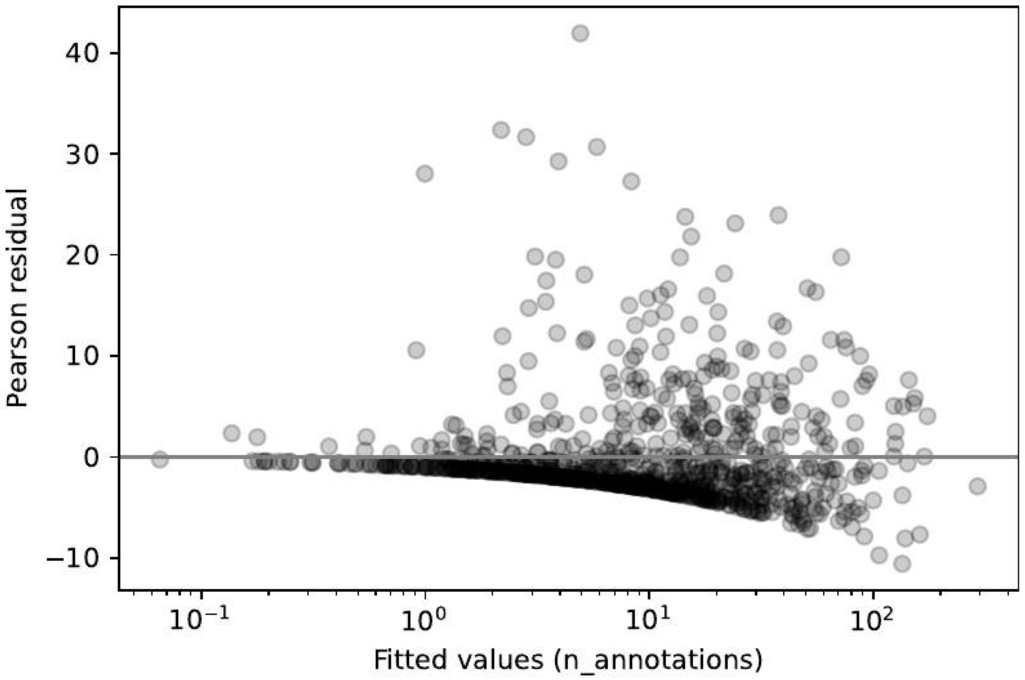

Figure S2. Pearson residuals versus predicted evening twilight detection count for Poisson GLM of detections against (in order of effect size); elevation, maximum daily temperature, day length, maximum wind speed, study year, temperature range, 9 am relative humidity, latitude, minimum temperature, and rainfall.

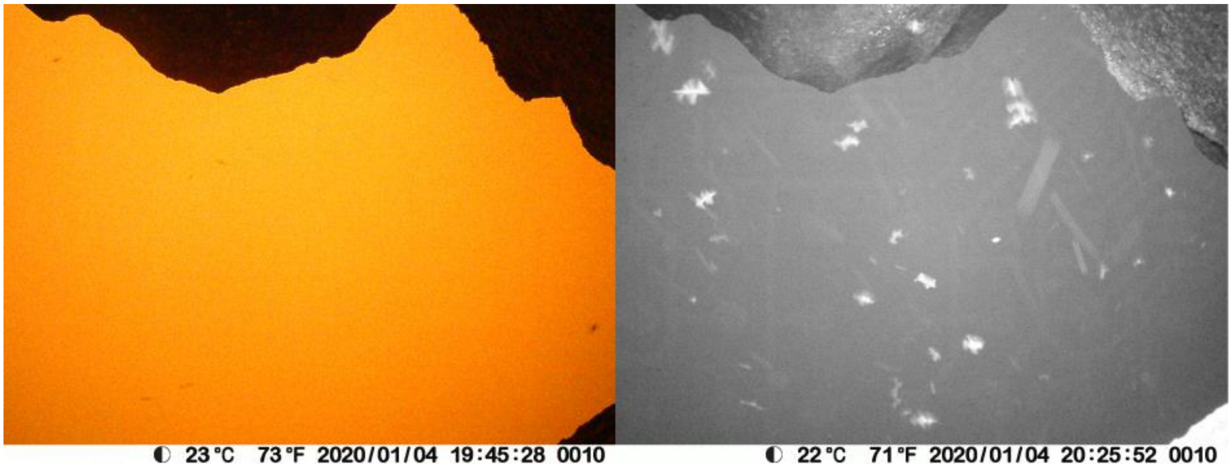

Figure S3. Bogong moths flying during bushfire outside aestivation cave near the top of Ken Green Bogong on January 4th 2020. **Left:** Photograph taken by camera, shortly before switching to "night mode". The air is thick with smoke, leading to the orange colour. Dark specks in the air are likely to be Bogong moths. **Right:** Photograph taken by the same camera, once it had switched to "night mode", with infra-red flash. Flying Bogong moths are clearly visible.

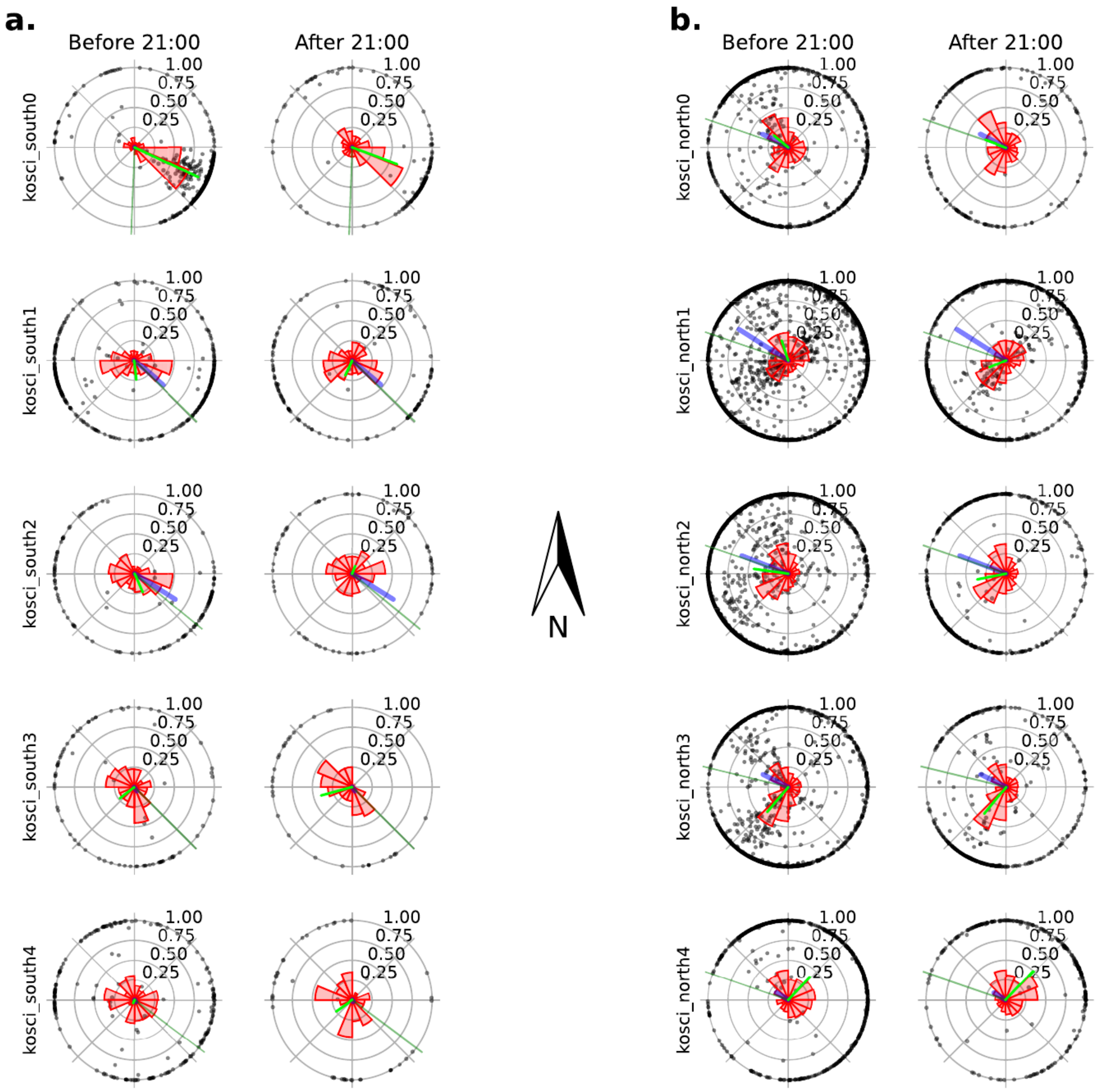

Figure S4. Trajectories of detected insects during (before 21:00 AEDT) and after nautical twilight for both transects. *Columns* indicate time period and *rows* indicate location. **a.** kosci_south transect. **b.** kosci_north transect. *Black dots*: Track (direction of displacement) of detected insect trajectories. *Radius* indicates the straightness of the trajectory, calculated as distance travelled divided by displacement (in pixel units). *Red bars*: Circular histogram of detected insect trajectories. The *bars* are equiareal (area—not height—indicates proportion of detections contained within each bin). *Lime green line*: Mean track (direction of displacement) of detected insect trajectories. *Radius* indicates circular mean vector length (with values closer to one indicating more concentrated tracks). *Blue line*: Fall line of the slope at the position of the camera. The direction indicates the direction of maximum gradient (perpendicular to topographic lines), and the radius indicates the gradient itself. *Dark green line*: Indicates the bearing of the base of the camera.

S2 Supplementary Tables

Table S1: Circular distribution models and corresponding output parameters selected using Akaike's information criterion (AIC, Akaike, 1973)

Loc.	Model	φ_1	κ_1	λ	φ_2	κ_2	θ
north0	5B	5.782	16.056	0.365	3.286	0.584	1.993
north1	5B	3.941	6.111	0.302	0.234	1.103	1.972
north2	5B	6.001	2.079	0.490	3.965	3.589	2.037
north3	5B	5.960	0.628	0.487	3.592	13.697	2.303
north4	5B	5.929	8.383	0.250	1.342	1.166	1.493
south0	2B	2.019	46.502	0.5†	-	0†	6.148
south1	5B	1.859	2.235	0.547	4.457	5.203	6.189
south2	5B	1.870	9.389	0.320	4.567	0.642	6.255
south3	5B	2.826	31.053	0.265	5.060	1.037	0.045
south4	5A	2.356	1.920‡	0.495	5.263	1.920‡	0.292

Models and parameters were computed using the CircMLE R package (Fitak and Johnsen, 2017) on flying insect detections at the respective camera locations (Loc.; for brevity, "kosci_" prefixes are removed from each location name). Model selection was performed on the models defined by Schnute and Groot (1992). Models appearing in table: 2B = "symmetric modified unimodal", 5A = "homogeneous bimodal", 5B = "bimodal". Models are mixtures of von Mises distributions with two components i ($i = 1{,}2$). φ_i denotes the mean direction of component i (in radians), κ_i the von Mises concentration parameter of component i, and λ the proportion assigned to the first component. θ is the azimuth of the summit of Mt. Kosciuszko (the nearest and highest peak) from the respective location. †Parameter fixed by model ($\lambda = 0.5$, $\kappa_2 = 0$). ‡Concentration parameters are assumed equal by model ($\kappa_1 = \kappa_2$).

Table S2. Model selection table for track directions relative to the azimuth of the summit of Mt. Kosciuszko.

Model	φ_1	κ_1	λ	φ_2	κ_2	ΔAIC
5B	2.055	0.741	0.654	4.480	4.584	0
5A	4.463	1.650	0.561	1.899	1.650‡	385.854
4B	4.466	5.483	0.301	1.325‡	0.393	693.199
2C	4.369	7.320	0.251	-	0†	897.322
3B	4.549	2.412	0.5†	1.408‡	0.987	1012.238
4A	4.607	1.429	0.582	1.465‡	1.429	1238.613
2A	3.645	0.410	1†	-	-	1368.769
3A	1.537	1.483	0.5†	4.679	1.483	1387.302
2B	3.851	0.802	0.5†	-	0†	1442.646
1	-	0†	1†	-	-	2274.647

†Parameter fixed by model. ‡Parameter depends on another parameter in model (i.e. $\varphi_2 = \varphi_1 + \pi(\mod 2\pi)$, or $\kappa_1 = \kappa_2$). Models for each location are sorted by the model selection criterion, ΔAIC. All other parameters follow the conventions of Table S1.

S3 References

Akaike, H., 1973. Maximum likelihood identification of Gaussian autoregressive moving average models. *Biometrika* 60.2, 255–265.

Fitak, R.R. and Johnsen, S., 2017. Bringing the analysis of animal orientation data full circle: model-based approaches with maximum likelihood. *J. Exp. Biol.* 220.21, 3878–3882.

Schnute, J.T. and Groot, K., 1992. Statistical analysis of animal orientation data. *Animal behav*. 43.1, 15–33.
